# Sexting among peruvian adolescents

**DOI:** 10.1186/1471-2458-14-811

**Published:** 2014-08-07

**Authors:** Joshua H West, Cameron E Lister, P Cougar Hall, Benjamin T Crookston, Paola Rivera Snow, Maria Elena Zvietcovich, Richard P West

**Affiliations:** Department of Health Science, Brigham Young University, 229-D Richards Building, Provo, Utah 84602 USA; Medicina Humana, Universidad Nacional de San Antonio Abad del Cusco, Cusco, Peru; Department of Special Education and Rehabilitation Counseling, Utah State University, Logan, Utah USA

**Keywords:** Sexting, Adolescent health, Peru, Sexual health, Risk behaviors

## Abstract

**Background:**

Sexting (sexual messaging via mobile devices) among adolescents may result in increased risky sexual practices, psychological distress and in some cases, suicide. There is very little research on sexting in developing nations, such as Peru. In particular, little is known about gender differences in the correlates of sexting. The purpose of this study was to determine the sexting prevalence and correlates of sexting among adolescent boys and girls in Cusco, Peru.

**Methods:**

The study sample comprised 949 high school aged adolescents from Cusco, Peru. Adolescents responded to questions about demographics, sexting behavior, and risk/protective factors. Separate regression models were constructed to compare correlates of sexting for boys and sexting for girls.

**Results:**

Twenty percent of the sample reported engaging in at least one instance of sexting. Boys reported higher rates of sexting than girls (35.17% vs. 13.19%, p = 0.000). Significant correlates for girls’ sexting included having been cyberbullied and parental factors. For boys, hypertexting, fighting, parental factors, and parental rules about sexting were significant.

**Conclusions:**

Peruvian health officials with an interest in reducing the effects of sexting among adolescents may choose to target boys differently than girls. These efforts may include advising parents to set clear rules and expectations about sexting and the appropriate use of mobile devices.

## Background

### Prevalence of sexting behaviors

Sexual messaging via mobile devices (sexting) is increasing among adolescents [1]. Recent reports of the prevalence of this phenomenon have varied, due primarily to differing definitions of sexting and age differences in study participants [2]. Sexting generally includes the use of mobile devices to send or receive sexually explicit text messages, images, or video [3]. The National Campaign to Prevent Teen and Unplanned Pregnancy published the first report of sexting prevalence in the United States. The *Sex and Tech Survey* included teens (13–19) and young adults (20–26) in its sample and online postings in its definition of sexting. Twenty-percent of teens reported they had sent or posted sexual images (either semi-nude or fully nude images of themselves) online or via a cell phone [4]. However, when asked about sending sexually suggestive messages via text message only (sexts), 39% of all teens reported sending sexts and 48% reported having received sexts [4]. An Associated Press-MTV Poll of 1,247 young people reported that 24% had sent sexually explicit images via their cell-phone or posted them online [5]. Specifically related to mobile technology/cell phone sexting, a 2009 report from Pew Internet reported that 4% of teens aged 12–17 had sent sexual images via mobile technology, and around 15% received them [6]. Taking into account these various definitions and sample demographics, rates of sexting behavior among U.S. teens range from 9% to 32% [7–11].

### Risk factors associated with sexting

Sexting may lead to serious mental health outcomes, such as emotional and psychological distress [12, 13], including suicide [14]. Additionally, female teens have reported feeling significant discomfort and anxiety from being asked by males to send sexts [11].

Sexting is associated with increases in both risky sexual behaviors and substance abuse among college students [15]. In a study by Benotsch, Snipes, Martine and Bull, 44% of 763 US college students reported having engaged in sexting, which was found to be associated with elevated levels of recent high risk sexual behaviors, specifically the number of sexual partners and the occurrence of unprotected sex [16]. Benotsch et al. also found sexting among college students to be associated with increases in substance abuse [16]. A similar study by Dake, Price, Maziarz, and Ward of 1279 middle school and high school students, aged 12–18 years, found sexting to be associated with risky sexual behavior and emotional health problems, including suicide attempts [7]. Dake et al. also identified correlations between sexting and recent substance use, including marijuana and cigarette use and binge drinking in the 30 days [7].

### Peru: Trends of sexual behavior and technology use

There are more youth today in Peru, aged 10–19 years, than there have been at any other time in the country’s history, making this a particularly important group to study [17]. The Global School Health Survey (GSHS) reports that 19.7% of youth in Peru, aged 13–17 years, have had sexual relations. Among those youth who reported sexual relations during the preceding month, only 64.3% used a condom [18]. Based on nationwide data, 89% of urban youth in Peru have access to mobile phones, while 46% have access to a computer, 26% to the Internet, and 43% to an MP3 player or iPod [19]. A significant proportion of youth who access the Internet use social media like Facebook (76%) and gain access through internet cafes [19], where parental oversight is minimal. Due to the pervasive use of mobile technologies among Peruvian adolescents, and the prevalence of sexual activity in this age group, we believe it is important to understand the role sexting may play in these behaviors. This paper is the first, according to our knowledge, to examine sexting behaviors of adolescents in Peru. Specifically, the purposes of this investigation were to determine the prevalence of sexting among a sample of adolescents in Cusco, Peru and to identify the correlates of sexting among adolescent boys and girls.

## Method

### Design and sample

The current study involved a cross sectional, convenience sample comprising 949 high school aged adolescents from the Cusco region of Peru. Respondents were aged 12–18 years and attended a public or private high school. Respondents represented all-boy, all-girl, and mixed gender high schools.

### Procedure

The researchers invited the participation in this study of all the public and private high schools in the Cusco region by first approaching, in person, the schools’ administrators. Fifty-three percent of the administrators granted permission for their school to participate. Once a school was included in the study, we made two additional visits, the first to recruit study participants and to distribute parental consent forms, and the second to collect consent forms and distribute the paper-pencil survey, which completed on the spot during the second visit. Approximately 1,000 students declined to participate. Reasons for nonparticipation included lack of parental consent, child assent, or being absent on the day of the survey. We wrote the survey questions first in English and then a native-Spanish speaker translated them into Spanish. Next, we back-translated the questions into English to ensure that the original intent of the questions was retained. We pilot tested the resulting study measures with 21 adolescents in the Cusco region and modified the questionnaires according to their feedback. The Brigham Young University Institutional Review Board approved all study materials and procedures. We included in the analysis only data from respondents for whom we received parental consent. We collected the completed surveys from each of the schools, entered the data from the surveys into an electronic database, and reviewed the database to identify outliers and correct possible data entry errors. Lastly, we offered a follow-up consultation at each participating school to examine the results from their school and discuss specific risk and protective factors related to technology and health behaviors.

### Measures

We asked study participants to report their age, race/ethnicity, income level, parental education, and year in school. We gathered information about sexting by asking the question, “On average, how much time per day do you spend sending or receiving sexually-related text messages?” The response categories included 0 minutes, 1–5 minutes, 5–30 minutes, 30–60 minutes, or more than 1 hour. We dichotomized this variable in the following format: 0 = spends no time on sexual related text messaging; and 1 = spends at least 1 minute per day on sexually-related text messaging. Parental rules about sexting were measured by, “Do your parents have rules about sending or receiving sexually-related text messages (yes/no)?” The number of text messages sent was measured using, “Thinking about yesterday, how much time did you spend texting?” The response categories included: none; 0–5 minutes; 5–30 minutes; 30–60 minutes; or more than 60 minutes. Being a victim of cyberbullying was measured using the following question: “How often have you been bullied through a cellular telephone or on the Internet?” The response categories included: not at all in the last 2 months; once or twice; a couple of times in the past month; once a week; or several times a week. This variable was dichotomized to compare individuals who have reported no cyberbullying in the past 2 months and those who reported being bullied at least once or twice. Academic achievement was measured by asking participants to report their average scores in classes. Response categories included: 91-100%; 81-90%; 71-80%; 65-70%; and 0-64%. Participation in fights was measured using the following question: “During the last 12 months, how many times have you participated in a physical fight?” The corresponding response categories included: 0; 1 time; 2–3 times; 4–5 times; 6–7 times; 8–9 times; 10–11 times; or greater than 12 times. Parents’ feelings about the adolescent’s sexual relations were measured by asking respondents, “How badly would your parents feel if you had sexual relations?” The response categories included: very bad; bad; not bad; and absolutely not bad at all.

### Analysis

We conducted an analysis of the data using Stata version 12.1 for Mac (StataCorp LP). We computed statistics to describe the study sample. We compared rates of potential factors associated with sexting between boys and girls using Chi-square test statistics. Adjusted logistic regression analysis was used separately for boys and girls to explore potential factors associated with sexting.

## Results

The majority of respondents were female (65.7%). The most common responses for “race” were: mestizo (48.8%) and Moreno (21.8%). Most respondents attended a mixed gender (54.8%) public school (62.2%). Just over 20% of the sample reported engaging in sexting (Table 1).

**Table 1:** **Descriptive statistics of the study sample (N = 949)**

Variable	Category	N	%
Gender			
	Male	326	34.35
	Female	649	65.65
Race/Ethnicity			
	Indigenous	27	3.00
	Moreno	196	21.78
	Mestizo	439	48.78
	Mixed	77	8.56
	Afro-Bolivian	5	0.56
	White	133	14.78
	Black	1	0.11
	Asian	22	2.44
Type of School			
	Private	361	37.76
	Public	595	62.24
Gender of Student-body			
	All boy	76	8.03
	All girl	352	37.17
	Mixed gender	519	54.80
Age			
	12	31	3.33
	13	115	12.37
	14	263	28.28
	15	274	29.46
	16	177	19.03
	17+	70	7.53
Sext			
	Yes	179	20.48
	No	695	79.52

*Note:* due to some instances of missing data, some categories may not be equal to the total sample of respondents.

A comparison of boys and girls (Table 2) revealed that boys engaged in significantly more sexting than girls (35.17% vs. 13.19%; chi = 57.0565; p = 0.000). Girls reported higher rates of parent disappointment for engaging in sex (p = 0.000). Girls also reported higher rates of parental rules about sexting (p = 0.001). Boys reported more fighting during the past 12 months (p = 0.000).

**Table 2:** **Chi-square comparison of potential factors associated with sexting between boys and girls (N = 917)**

Cyberbullied in the past 12 months		Boy		Girl		Stat	Sig
	Yes	60	19.11	131	21.72	0.8572	0.355
	No	254	80.89	472	78.28		
Number of fights in the past 12 months							
	0	112	34.78	490	78.90	213.4755	0.000
	1-2	79	24.53	85	13.69		
	3-4	77	23.91	23	3.70		
	5-6	23	7.14	10	1.61		
	7-8	7	2.17	9	1.45		
	9-10	7	2.17	2	0.32		
	10-11	2	0.62	0	0.00		
	12+	15	4.66	2	0.32		
Parents would be disappointed if you had sex							
	Very bad	121	39.80	499	85.15	210.9575	0.000
	Bad	55	18.09	47	8.02		
	Not bad	61	20.07	25	4.27		
	Absolutely not bad at all	67	22.04	15	2.56		
Sexting							
	Yes	102	35.17	76	13.19	57.0565	0.000
	No	188	64.83	500	86.81		
Parents have rules about sexting							
	Yes	119	41.72	288	53.53	10.3377	0.001
	No	166	58.25	250	46.47		
# text messages sent yesterday							
	0-5 minutes texting	183	60.60	378	65.51	3.2839	0.350
	5-30 minutes texting	63	20.86	93	16.12		
	30-60 minutes texting	18	5.96	33	5.72		
	More than 60 minutes texting	38	12.58	73	12.65		
Academic Achievement							
	0-64%	17	5.28	46	7.46	3.6791	0.451
	65-70%	77	23.91	163	26.42		
	71-80%	138	42.86	262	42.46		
	81-90%	79	24.53	126	20.42		
	91-100%	11	3.42	20	3.24		

*Note:* due to some instances of missing data, some categories may not be equal to the total sample of respondents.

Using a multivariate approach, for girls, having been cyberbullied resulted in an increased odds of sexting (OR = 2.019; p = .022) as did having parents feeling less badly about the respondent having sexual relations (OR = 1.799; p = .000) (Table 3). For boys (Table 4), factors associated with increased odds for sexting included sending more text messages during the previous day, parents feeling less badly about the respondent having sexual relations (OR = 1.399; p = .014), and having been involved in fighting during the past 12 months (OR = 1.313; p = .003). Having parents with rules about sending or receiving sexual messages was also associated with decreased odds of sexting for boys in this study sample (OR = 0.519; p = .045).

**Table 3:** **Logistic regression, factors associated with sexting among girls (N = 459)**

					95% CI	
Variable	OR	Std. Err.	Z	P	Low	High
Parent rules about sexting	.6433001	.1850028	−1.53	0.125	.3661185	1.130331
Cyber-bullyied	2.018929	.6183585	2.29	0.022	1.107681	3.679827
Grade	1.106321	.1550271	0.72	0.471	.8406272	1.455991
Parents feel bad if sexually active	1.799322	.2985303	3.54	0.000	1.299822	2.490773
Number of fights in the past 12 months	1.218819	.1768804	1.36	0.173	.9170836	1.619829
Academic acheivement	.8248141	.1292042	−1.23	0.219	.606762	1.121227
0-5 minutes texting	-	-	-	-	-	-
5-30 minutes texting	1.676044	.6198025	1.40	0.163	.8119148	3.459876
30-60 minutes texting	2.137278	1.12558	1.44	0.149	.7613466	5.999838
More than 60 minutes texting	1.243663	.5374858	0.50	0.614	.5331268	2.90118
Cons	.0568369	.0405236	−4.02	0.000	.0140521	.2298901

**Table 4:** **Logistic regression, factors associated with sexting among boys (N = 230)**

					95% CI	
Variable	OR	Std. Err.	Z	P	Low	High
Parent rules about sexting	.5188538	.1698481	−2.00	0.045	.2731506	.985571
Cyber-bullyied	.8749075	.3526317	−0.33	0.740	.3970865	1.927698
Grade	1.202662	.1796177	1.24	0.217	.8974626	1.611649
Parents feel bad if sexually active	1.398703	.1913362	2.45	0.014	1.069757	1.8288
Number of fights in the past 12 months	1.312535	.11811	3.02	0.003	1.100309	1.565695
Academic achievement	1.153725	.2094005	0.79	0.431	.8083691	1.646626
0-5 minutes texting	-	-	-	-	-	-
5-30 minutes texting	2.453829	.9294675	2.37	0.018	1.167949	5.155426
30-60 minutes texting	4.803865	3.137205	2.40	0.016	1.335692	17.27728
More than 60 minutes texting	3.004522	1.408366	2.35	0.019	1.198881	7.529653
Cons	.0357671	.0301718	−3.95	0.000	.0068461	.1868638

## Discussion

The current study reports texting rates in Cusco similar to those found in U.S. studies of teen sexting, particularly the Sex and Tech Survey conducted by the National Campaign to Prevent Teen and Unplanned Pregnancy [4]. Factors associated with sexting were generally different for girls and boys. Factors for girls ranged from being cyberbullied and parental views of adolescent sexual activity, while factors for boys included fighting, excessive texting, parental views of adolescent sexual activity and parental rules about sexting.

Previous studies have yielded mixed results related to the impact of gender on texting rates. The current study found a large gender disparity in sexting, with nearly three times as many boys sexting compared to girls. One possible explanation for the difference between male and female sexting rates is the forwarding behavior among teenage males, who have been found to be more likely to forward images received than females [10, 14]. This does not explain, however, the comparatively large gender disparity among the current study of Peruvian youth and previous studies among U.S. youth. Examining how culturally-specific gender differences impact sexting behaviors merits further investigation.

Consistent with previous studies exploring correlates of risk behaviors with sexting [20], the current study found those who text more, also sext more [7]. Inasmuch as boys and girls did not differ in this study on the amount of daily texting, the finding that boys’ *hypertexting* was associated with higher rates of sexting may relate to gender differences relating to the purpose of texting. Reid & Reid (2004) concluded that boys use texting largely to coordinate activities and are comfortable communicating to a group [21] whereas girls’ use of texting is related more to relationship-oriented conversations. These authors contend that to completely embrace the technology for communicating, girls require a greater understanding of the nature of the network. For example, trust in the recipient that he or she will not share the contents with others. Higher rates of sexting among *hypertexting* boys in this study may result from boys’ overall comfort with using texting for communicating a variety of topics, including those that might be sexually explicit. Hence, monitoring sexting and excessive texting, or *hypertexting*, may help parents and professionals identify adolescents at risk and provide opportunities for intervention. In other words, gender differences in texting seem to be similar to those in other forms of communication, such as conversation and non-mediated writing. The concern, however, is that boys’ communication patterns contain more sexually-oriented content and could lead to more sexual activity.

Parents’ influence in preventing adolescent risk behaviors is often undervalued [22–24] in favor of presumed-to-be-stronger peer and school influences. However, some studies report that parenting practices account for as much as 40% of the variance in adolescents’ risk behaviors [22, 24, 25]. One example of parental influence that is protective relates to parent-adolescent communication about sexuality, which is generally associated with a decrease in sexual risk [26]. Parents who clearly communicate expectations related to sexuality are most influential in supporting their youth’s healthy approach to sexuality, especially if expectations are accompanied with monitoring and support [27]. Unlike Huebner & Howell, who found no significant differences between boys and girls in their communication with parents about sexual health, girls in this study were more likely to report that they perceived their parents would be disappointed if they had sex. Gender differences of this nature have not previously been documented in a sexting context. These findings may have cultural underpinnings whereby gender role socialization may vary by gender in many families of Latino heritage [28]. Insomuch as boys appear most influenced by clear communication related to sexuality, which relates to sexting, parents of Peruvian adolescent males may wish to establish or strengthen lines of communication in an effort to reduce or prevent risk behaviors of this nature.

Being in a physical fight in the past 12 months was associated higher rates of sexting in this study. Fighting among Peruvian youth is common, with 52.4% of boys and 21.5% of girls, aged 13–15 years, reporting having been in a fight one or more times in the past 12 months [18]. Prolonged exposure to violence in general, and fighting or physical assault in particular, has been shown to be correlated with the adoption of other health risk behaviors, including early initiation of sexual behaviors [18]. Other sociocultural factors, such as risk-taking and traditional gender roles, likely also influence increased male risk-taking behaviors, which may explain the association with sexting and fighting among males in this study. Risk-taking has long been associated with the masculine psyche as explained by the “Risk as Value” hypothesis which forecasts increased male risk-taking based upon socially-instilled beliefs that risk-taking is a highly valued masculine tendency [29]. Traditional male gender roles in Peru, which have been described both positively and negatively as *machismo* and *caballerismo,* are examples of such socially-instilled beliefs in Peru. Risk-taking behaviors such as fighting and sexting appear to provide perceived social value and reward among contemporary Peruvian adolescent males [17].

Evolutionary psychology examines, among other phenomena, how psychological adaptations to environment can help explain human behavior. One area of interest in evolutional psychology is mate value, which Fisher, et al. broadly defined as “the total sum of characteristics an individual possesses at a given moment and within a particular context that impacts on their ability to successfully find, attract, and retain a mate [30] p.157.” Self-perceived mate value, a largely socioculturally constructed measurement of an individual’s appeal to potential mates is associated with both early sexual debut and increased sexual risk-taking [31]. Correlations between fighting and sexting found in this study may be predictive of a positive social standing and mate value or motivated by a desire to increase social standing and mate value. Additional physiological factors likely play a role in both fighting and sexting among males in general, and Peruvian boys in the current study in particular. Although the role of testosterone in risk-taking and aggressive behaviors remains a topic of ongoing investigation, there is evidence to suggest that the male sex hormone plays a significant role in explaining a variety of male gender-specific risk behaviors [32–34]. Associated with both libido and patterns of physical aggression, testosterone provides one explanation for associations between sexting and fighting among males in the current study. Further research exploring potential sociocultural and physiological factors related to sexting is warranted.

While national and international efforts to improve health outcomes for Peruvian youth are robust, coordinated school health education efforts at the schools participating in this study were nonexistent. School administrators were eager to work with us on the current study and showed considerable concern for the health of students. With a recent focus on improving academic outcomes through health promotion efforts from both the Ministry of Education and the Ministry of Health, school-based health programs and instruction may be a not-so-distant reality for Peruvian schools. In the meantime, Peruvian schools can communicate to parents the need for establishing clear guidelines for their children related to the use of technology in general and sexting in particular. Schools and teachers can similarly work to implement well-established practices for increasing protective factors such as establishing caring relationships with students, maintaining high expectations for student performance, and providing students a variety of opportunities for participation and contribution in the classroom, school, and community [35].

### Limitations

Interpreting the findings of this study involves several considerations. First, data for this study came from respondents’ self-reports which may be influenced by social desirability. Questions that were highly personal or potentially embarrassing in some way may have caused respondents to respond different than reality. Efforts to minimize this impact included the adaptation of established measures from previous studies that have shown little variability in respondents’ reports. Second, these findings may or may not be representative of all Peruvian adolescents or adolescents in the Cusco region. Data were collected from respondents at schools that agreed to participate in this study, which may limit to some extent the external validity of the study findings. A balance of private, public, all-female, and all-male schools was targeted to at least provide wide representation. Future studies of this nature may benefit from more strategic sampling methodologies in order to minimize this concern.

## Conclusion

Peruvian adolescents currently engage in sexting behaviors; boys reporting higher rates than girls. Efforts to prevent sexting in this population may be successful with parental involvement to clearly communicate family or household rules and expectations. These efforts should be tailored to the gender of the target adolescent, and should target hypertexting, fighting and issues related to sexuality. This study is one of the first in a developing setting to identify gender differences in the prevalence of and correlates of sexting among adolescents. The results from this study will be useful in prevention and intervention efforts targeting adolescents to avoid the negative effects of sexting.

## Authors’ information

BC, JW, and PH are assistant professors in the Department of Health Science at Brigham Young (BYU) University. CL is a Master of Public Health Student at BYU, and PS is a graduated Master of Public Health student from BYU. MZ is a professor of Medicina Humana, Universidad Nacional de San Antonio Abad del Cusco. RW is a professor in the Department of Special Education and Rehabilitation Counseling, at Utah State University.

## Abbreviations

GSHS: Global school health survey
OR: Odds ratio.

## References

[1] O’Keeffe GS, Clarke-Pearson K (2011). The impact of social media on children, adolescents, and families. Pediatrics.

[2] Lounsbury K, Mitchell K, Finkelhor D (2011). The true prevalence of “sexting.”.

[3] Chalfen R (2009). “It”s only a picture’: sexting, “smutty”snapshots and felony charges. Vis Stud.

[4] *Sex and tech: results from a survey of teens and young adults*. [http://www.thenationalcampaign.org/sextech/pdf/sextech_summary.pdf]

[5] *Digital abuse survey*. 2009. [http://surveys.ap.org/data/KnowledgeNetworks/AP_Digital_Abuse_Topline_092209.pdf]

[6] Lenhart A (2009). Teens and sexting. A Pew Intern Am Life Proj Rep.

[7] Dake JA, Price JH, Maziarz L, Ward B (2012). Prevalence and correlates of sexting behavior in adolescents. Am J Sex Educ.

[8] Mitchell KJ, Finkelhor D, Jones LM, Wolak J (2012). Prevalence and characteristics of youth sexting: a national study. Pediatrics.

[9] Rice E, Rhoades H, Winetrobe H, Sanchez M, Montoya J, Plant A, Kordic T (2012). Sexually explicit cell phone messaging associated with sexual risk among adolescents. Pediatrics.

[10] Strassberg DS, McKinnon RK, Sustaíta MA, Rullo J (2013). Sexting by high school students: An exploratory and descriptive study. Arch Sex Behav.

[11] Temple JR, Paul JA, van den Berg P, Le VD, McElhany A, Temple BW (2012). Teen sexting and its association with sexual behaviors. Arch Pediatr Adolesc Med.

[12] Gordon-Messer D, Bauermeister JA, Grodzinski A, Zimmerman M (2012). Sexting among young adults. J Adolesc Heal.

[13] Sadhu JM (2012). Sexting: The impact of a cultural phenomenon on psychiatric practice. Acad Psychiatry.

[14] Curnutt H (2012). Flashing your phone: Sexting and the remediation of teen sexuality. Commun Q.

[15] Dir AL, Cyders MA, Coskunpinar A (2013). From the bar to the bed via mobile phone: A first test of the role of problematic alcohol use, sexting, and impulsivity-related traits in sexual hookups. Comput Human Behav.

[16] Benotsch EG, Snipes DJ, Martin AM, Bull SS (2013). Sexting, substance use, and sexual risk behavior in young adults. J Adolesc Heal.

[17] Bayer AM, Cabrera LZ, Gilman RH, Hindin MJ, Tsui AO (2010). Adolescents can know best: Using concept mapping to identify factors and pathways driving adolescent sexuality in Lima. Peru Soc Sci Med.

[18] GSHS (2010). Encuesta global de salud escolar: resultados Peru.

[19] AudienceScapes: *New Media Use Among Peru’s Youth and Young Adults*. [http://www.audiencescapes.org/country-profiles-urban-peru-article-urban-youth-new-media-use]

[20] Johnson TD (2011). Excessive texting, social networking linked to health risks for teenagers. Nations Heal.

[21] Reid FJM, Reid DJ (2004). Text appeal: the psychology of SMS texting and its implications for the design of mobile phone interfaces. Campus-Wide Inf Syst.

[22] Patterson GR, DeBaryshe BD, Ramsey E (1989). A developmental perspective on antisocial behavior. Am Psychol.

[23] Patterson GR, Reid JB, Dishion TJ (1992). Antisocial boys. Castalia Pub Co.

[24] Walker HM, Colvin G, Ramsey E (1995). Antisocial behavior in school: Strategies and best practices.

[25] Tolan PH, McKay MM (1996). Preventing serious antisocial behavior in inner-city children: An empirically based family intervention program. Fam Relat.

[26] Hutchinson MK, Cooney TM (1998). Patterns of parent-teen sexual risk communication: Implications for intervention. Fam Relat.

[27] Huebner AJ, Howell LW (2003). Examining the relationship between adolescent sexual risk-taking and perceptions of monitoring, communication, and parenting styles. J Adolesc Heal.

[28] Raffaelli M (2001). Ontai LL: **“She’s 16 years old and there’s boys calling over to the house”: An exploratory study of sexual socialization in Latino families**. Cult Heal Sex.

[29] Byrnes JP, Miller DC, Schafer WD (1999). Gender differences in risk taking: A meta-analysis. Psychol Bull.

[30] Fisher M, Cox A, Bennett S, Gavric D (2008). Components of self-perceived mate value. J Soc Evol Cult Psychol.

[31] James J, Ellis BJ, Schlomer GL, Garber J (2012). Sex-specific pathways to early puberty, sexual debut, and sexual risk taking: Tests of an integrated evolutionary–developmental model. Dev Psychol.

[32] Rowe R, Maughan B, Worthman CM, Costello EJ, Angold A (2004). Testosterone, antisocial behavior, and social dominance in boys: pubertal development and biosocial interaction. Biol Psychiatry.

[33] Popma A, Vermeiren R, Geluk C, Rinne T, van den Brink W, Knol DL, Jansen L, van Engeland H, Doreleijers T (2007). Cortisol moderates the relationship between testosterone and aggression in delinquent male adolescents. Biol Psychiatry.

[34] Vermeersch H, T’sjoen G, Kaufman J-M, Vincke J (2008). The role of testosterone in aggressive and non-aggressive risk-taking in adolescent boys. Horm Behav.

[35] Benard B (2002). From risk to resiliency: what schools can do.

[CR36] The pre-publication history for this paper can be accessed here:http://www.biomedcentral.com/1471-2458/14/811/prepub

